# Surgery

**Published:** 1906-09

**Authors:** T. Carwardine


### SURGERY.

The progress made in the methods and application of **gastro-enterostomy** in the last decade has been one of the most marked features in surgery. Its mortality is now no longer formidable; its utility is sought at earlier stages of disease; and, with established principles accepted, its advances are chiefly in the direction of improvements in the mechanical details. The anterior operation is now reserved for exceptional cases, the posterior operation being generally the better in its results; the "loop" method seems destined to disappear on account of the frequency of acute "vicious circle"; and the recent improvements in the "no loop" method of gastro-enterostomy chiefly concern the direction and position of the opening between the stomach and intestine.

The pioneer work and experience of the brothers Mayo are valuable, and a *résumé* is published in two recent papers by Dr. W. J. Mayo, with an analysis of some five hundred cases.[1] Their early cases showed a particularly high mortality, but they have recently done over a hundred cases with only one death. Their general mortality for the whole series was 6 per cent. for benign and 19 per cent. for malignant cases. The paper passes in review the various methods the Mayos have employed, till finally deciding that the operation of choice is without a loop, as practised by Czerny for years, and employed by the writer since 1903; and the following remarks apply entirely to the posterior "no loop" method of gastro-enterostomy.

To properly appreciate the advantages of this method, some physiological and anatomical facts must be understood. When empty, the lowest part of the stomach is near the pylorus, and nearly in the middle line of the body. As the stomach distends, it becomes more horizontal, the pylorus passes to the right, and the greater curvature insinuates itself between the layers of the gastro-colic omentum so as to become apposed to the gastro-epiploic artery, which is situated three-quarters of an inch away from the greater curvature when the stomach is empty. The lesser curvature is more fixed, and consists of a longer vertical part and a shorter horizontal pyloric portion, with a concavity or angle between them. Now the right hand margin of the gastro-jejunal opening should be placed opposite this angle formed by the two portions of the lesser curvature, the remainder of the opening being to the left thereof. Formerly the writer made the right hand margin the lowest point also, the jejunum being rotated so that the line of its peristalsis agreed with that of the stomach; but, as we shall describe later on, the rotation of the jejunum is no longer carried out, and the left-hand margin of the aperture is made the most dependent point, by separating the omentum from the greater curvature, pushing the gastro-epiploic vessel out of the way, and pulling out one-fourth of an inch of the anterior wall posteriorly. By such "no loop" operation there is no kink or bend as with the "loop" operation, and there is no marked side channel in which the food on the one hand, or the bile and pancreatic juice on the other hand, will collect. Peterson's point of election for the commencement of the incision in the bowel varies according to the ease of attachment from one to three inches from the origin of the jejunum.

Other points of note in this operation are the following: The abdominal incision is made through the rectus, three-quarters of an inch to the right of the middle line. The union of stomach to bowel is effected by silk suture externally, and hardened catgut internally. The margins of the incised meso-colon are sutured to the line of union.

In a supplementary article, based upon 136 operations

[1] *Ann. Surg.*, 1905, xlii. 641.

with but one death,[1] Mayo considers the common subsequent condition of chronic regurgitation of bile which comes on at intervals in a small percentage of patients, usually within ten weeks of the original operation; a condition which is found on re-operation to be due to partial kinking or twisting at the gastric attachment. In the cases of "no loop" operation in which this complication ensued, the anastomosis of the jejunum and stomach was made in the line of peristalsis, the proximal portion of the jejunum being attached to the posterior gastric wall to the left and above, and the distal end of the jejunum to the right and lower part of the stomach; and it seems that the complication referred to was due to this fact. The question therefore arises as to whether the idea of continuity of peristalsis has any practical significance. Now the natural disposition of the first part of the jejunum is to pass first into the left kidney pouch underneath the splenic flexure of the colon, thence into the left abdominal fossa; so that if it be twisted to conform to the line of gastric peristalsis the natural descent and direction are altered. The Mayos have abandoned the reversal of the jejunum in the last sixty-five cases of the posterior "no loop" operation without any subsequent trouble or death, and "with vastly better results." The steps of the operation are carried out in the usual way, the lowest part of the anastomosed stomach being a down-drawn part of the anterior wall towards the left. The anastomosis is made with the aid of light elastic curved holding clamps, preferably Doyen's, placed side by side; and union effected by an external layer of silk suture, and an internal layer of hardened catgut.

Some three years ago the writer reviewed the literature with reference to **malignant disease of the vermiform appendix**;[2] and an interesting and exhaustive paper was recently read by Dr. Rolleston and Mr. Lawrence Jones on this subject.[3] They have very critically examined all the reported cases, and find that there are some forty-two cases available for analysis as being authentic, viz.: carcinoma, thirty-seven cases; endothelioma, three cases; sarcoma, three cases. Considering the frequency of operations for appendicitis, primary malignant disease of the vermiform appendix is therefore extremely rare. The majority of cases have been reported by American authors, and to the above total I may add a third case of sarcoma of the appendix under my care, and not yet published.

The earliest report of a case found *post mortem* was by Merling in 1838,[4] quoted by Elting;[5] but as it was not accompanied by microscopical evidence, it must be regarded as doubtful. The first authentic case was reported by Beger[6] in a

[1] *Ibid.*, 1906, xliii. 537. [2] *Bristol M.-Chir. J.*, 1903, xxi. 252.
[3] *Med. Chir. Tr.*, 1906, lxxxix.
[4] *J. de l'Expérience*, 1838. [5] *Ann. Surg.*, 1903, xxxvii. 549.
[6] *Berl. klin. Wchnschr.*, 1882, xix. 616.

man 47 years of age, who for three and a half years had suffered from a fistula in the right iliac fossa, dating from an abscess in that situation, and who died shortly after operation. As an indication of the rarity of the affection, it may be mentioned that Maydl in 1883 recorded only one case in over twenty thousand autopsies, but without giving details.

The first recorded case of sarcoma was by Warren in 1898, and the second by Paterson in 1903. In 1900 there were nine genuine cases of malignant disease of the appendix recorded; so that, with increased watchfulness and more frequent operations on the appendix, there is a tendency to an increase in the number of cases noted. Moreover, following the trend of pathological opinion, several of the later cases have been reported as endothelioma. Of the forty-two cases, the nature of the disease was discovered after operation in thirty-three, and on *post-mortem* examination in nine; in no case was the true nature of the disease suspected before operation, although this suspicion existed in an unpublished case under my care.

It is remarkable that forty-one out of the forty-two cases have been placed on record during the last ten years, that in two cases the growth has been so small as to be only recognisable by the microscope, and that at St. George's Hospital during the last five years two appendices which had been excised for simple inflammation presented the change, as well as one found after death.

Carcinoma of the appendix appears to occur at an earlier average age than carcinoma of the intestine elsewhere, 75 per cent. of the cases having occurred before 40. This would suggest that inflammation of the appendix is a predisposing factor, more so than the presence of a concretion, which has only been found in two cases; evidences of acute or chronic inflammation were present in the majority of cases. Secondary growths were only observed to be present in five out of the total number. The commonest variety is a spheroidal-celled carcinoma, resembling the histological characters of multiple primary growths of the ileum and jejunum, or some forms of endothelioma, and raising a difficulty as to the exact pathological designation.

The clinical symptoms have been nearly always those of appendicitis, and in twenty-seven of the thirty-three cases treated operation was done for appendicitis. Metastases are the exception rather than the rule, and a free local removal may be followed by perfect immunity from recurrence, though in a few cases the operation has been more extensive. Some of the patients have been heard of years after operation free from recurrence. Therefore the immediate prognosis of operation is good, and there is a prospect of freedom from subsequent recurrence, particularly in the spheroidal-celled carcinomata or endotheliomata.

T. Carwardine.

